# Supplementary material for: Large scale paired antibody language models
Source: PLoS Comput Biol. 2024 Dec 6;20(12):e1012646. doi: 10.1371/journal.pcbi.1012646 (PMC11654935; doi:10.1371/journal.pcbi.1012646)
Supplement: S1 Appendix — Example code for the use of the IgT5(-unpaired) and IgBert(-unpaired) models, along with links to more detailed information. (PDF) [file pcbi.1012646.s001.pdf]

S1 Appendix. Model usage

IgBert can be used through the Hugging Face library as follows

```
from transformers import BertForMaskedLM, BertTokenizer
tokenizer = BertTokenizer.from_pretrained("Exscientia/IgBert",
                                          do_lower_case=False)
model = BertForMaskedLM.from_pretrained("Exscientia/IgBert")
```

Here `Exscientia/IgBert` can be replaced by `Exscientia/IgBert_unpaired` to load the unpaired model instead.

Similarly, IgT5 can be used with

```
from transformers import T5Tokenizer, T5Model
tokenizer = T5Tokenizer.from_pretrained("Exscientia/IgT5",
                                        do_lower_case=False)
model = T5Model.from_pretrained("Exscientia/IgT5")
```

where one can again replace the paired model `Exscientia/IgT5` by `Exscientia/IgT5_unpaired` to load the model pre-trained on unpaired sequences.

Note that for the IgBert model, paired sequences are combined by using the `[SEP]` separator token, while for the paired IgT5 model the corresponding separator is `</s>`.

More detailed usage examples can be found in each model card:

- IgBert: `https://huggingface.co/Exscientia/IgBert`
- IgBert-unpaired: `https://huggingface.co/Exscientia/IgBert_unpaired`
- IgT5: `https://huggingface.co/Exscientia/IgT5`
- IgT5-unpaired: `https://huggingface.co/Exscientia/IgT5_unpaired`

For most downstream task, we recommend using IgT5, which has shown the best performance across our benchmarks. For more ressource-constrained or high throughput applications, IgBert can provide almost comparable performance with a more limited memory footprint.
