# Supplementary material for: Large scale paired antibody language models
Source: PLoS Comput Biol. 2024 Dec 6;20(12):e1012646. doi: 10.1371/journal.pcbi.1012646 (PMC11654935; doi:10.1371/journal.pcbi.1012646)
Supplement: S2 Appendix — Overview of the training dataset, which consists of the OAS database accessed in November 2023. (PDF) [file pcbi.1012646.s002.pdf]

S2 Appendix. OAS dataset

Our training dataset consists of the OAS database, accessed in November 2023. This dataset contains both paired and unpaired antibody variable region sequences, which predominantly come from healthy human donors. All sequences contain the VDJ recombination, though some are missing a portion of the first framework region.

Table 1: Overview of the OAS dataset. We provide the number of sequences of heavy and light chains, as well as the breakdown of species that compose the dataset.

Origin	Unpaired sequences	Paired sequences
Heavy chains	2,069,765,758	2,038,528
Light chains	355,894,853	2,038,528
Human	2,242,576,269	1,954,079
Mouse	172,770,518	28,507
Rat	5,241,023	55,942
Rabbit	3,471,165	0
Camel	1,601,636	0
