# Supplementary material for: Large scale paired antibody language models
Source: PLoS Comput Biol. 2024 Dec 6;20(12):e1012646. doi: 10.1371/journal.pcbi.1012646 (PMC11654935; doi:10.1371/journal.pcbi.1012646)
Supplement: S3 Appendix — The sequence recovery achieved by each model on a test set of unpaired sequences. (PDF) [file pcbi.1012646.s003.pdf]

S3 Appendix. Unpaired sequence recovery

The sequence recovery achieved by each model on a test set of unpaired sequences is shown in Table 1. The IgT5-unpaired model tends to achieve the highest amino acid recovery in the CDR loops, while the IgT5 model has comparable accuracy in the framework.

Table 1: Fraction of correctly predicted residues by region, after masking 15% of the sequence for a random test set of 100k unpaired sequences. The best, second and third best performing models for each region are shown in bold, underlined and italic respectively.

Model	FWH1	FWH2	FWH3	FWH4	CDRH1	CDRH2	CDRH3	Total VH
AbLang	0.960	0.951	0.937	0.962	0.890	0.874	0.584	0.890
AntiBERTy	0.963	0.950	0.937	0.962	0.893	0.877	0.530	0.884
ProtBert	0.731	0.715	0.687	0.793	0.626	0.465	0.279	0.633
IgBert-unpaired	*0.967*	0.957	*0.951*	0.972	0.906	*0.898*	0.635	0.907
IgBert	0.967	*0.958*	0.950	*0.972*	*0.909*	0.898	*0.636*	*0.907*
ProtT5	0.784	0.816	0.870	0.892	0.718	0.627	0.346	0.756
IgT5-unpaired	**0.969**	**0.962**	<u>0.957</u>	<u>0.976</u>	**0.920**	**0.920**	**0.703**	**0.922**
IgT5	<u>0.968</u>	<u>0.960</u>	**0.957**	**0.977**	<u>0.918</u>	<u>0.918</u>	<u>0.699</u>	<u>0.921</u>
Model	FWL1	FWL2	FWL3	FWL4	CDRL1	CDRL2	CDRL3	Total VL
AbLang	0.923	0.924	0.928	0.909	0.795	0.811	0.765	0.899
AntiBERTy	0.941	0.920	0.927	0.910	0.816	0.803	0.777	0.903
ProtBert	0.628	0.744	0.741	0.587	0.421	0.413	0.255	0.627
IgBert-unpaired	0.942	*0.934*	0.938	0.930	0.830	*0.854*	*0.805*	0.916
IgBert	*0.942*	0.932	*0.940*	*0.932*	*0.833*	0.841	0.803	*0.916*
ProtT5	0.799	0.856	0.829	0.764	0.620	0.552	0.468	0.766
IgT5-unpaired	<u>0.945</u>	**0.944**	**0.946**	**0.944**	**0.865**	**0.884**	<u>0.841</u>	**0.929**
IgT5	**0.946**	<u>0.942</u>	<u>0.945</u>	<u>0.942</u>	<u>0.852</u>	<u>0.875</u>	**0.844**	<u>0.927</u>
