# Supplementary material for: Large scale paired antibody language models
Source: PLoS Comput Biol. 2024 Dec 6;20(12):e1012646. doi: 10.1371/journal.pcbi.1012646 (PMC11654935; doi:10.1371/journal.pcbi.1012646)
Supplement: S4 Appendix — The correlation of the predicted binding energy and expression with the data. (PDF) [file pcbi.1012646.s004.pdf]

S4 Appendix. Correlation for binding affinity and expression

We show the correlation of the predicted binding energy and expression with the data in Table 1, for a linear fit on a feature of averaged embedding representations.

Table 1: Pearson correlation for a linear model applied on the embeddings of each language model to predict binding or expression. The best, second and third best performing models for each benchmark are shown in bold, underlined and italic respectively.

Model	Binding $N = 422$	Binding $N = 2048$	Binding $N = 4275$	Expression $N = 4275$
AbLang	*0.556* $\pm$ 0.106	*0.499* $\pm$ 0.037	<u>0.496</u> $\pm$ 0.032	0.665 $\pm$ 0.022
AntiBERTy	0.501 $\pm$ 0.098	0.47 $\pm$ 0.059	0.449 $\pm$ 0.026	0.634 $\pm$ 0.027
ProtBert	0.472 $\pm$ 0.157	0.406 $\pm$ 0.043	0.342 $\pm$ 0.039	0.708 $\pm$ 0.021
IgBert-unpaired	0.549 $\pm$ 0.082	0.459 $\pm$ 0.057	0.435 $\pm$ 0.029	0.608 $\pm$ 0.024
IgBert	**0.636** $\pm$ 0.044	0.438 $\pm$ 0.03	0.457 $\pm$ 0.047	0.563 $\pm$ 0.078
ProtT5	0.56 $\pm$ 0.085	0.456 $\pm$ 0.059	*0.467* $\pm$ 0.04	**0.837** $\pm$ 0.012
IgT5-unpaired	<u>0.575</u> $\pm$ 0.087	<u>0.534</u> $\pm$ 0.053	0.457 $\pm$ 0.023	<u>0.76</u> $\pm$ 0.019
IgT5	0.554 $\pm$ 0.062	**0.566** $\pm$ 0.065	**0.51** $\pm$ 0.023	*0.75* $\pm$ 0.041
