# Supplementary material for: Large scale paired antibody language models
Source: PLoS Comput Biol. 2024 Dec 6;20(12):e1012646. doi: 10.1371/journal.pcbi.1012646 (PMC11654935; doi:10.1371/journal.pcbi.1012646)
Supplement: Attachment — Submitted filename: reviewer_LMpaper.pdf [file pcbi.1012646.s005.pdf]

We thank the reviewers for their careful consideration of our manuscript. We have made changes to the article in this revision, notably removing the section on perplexity and changing the discussion on catastrophic forgetting. Comments by the reviewers are addressed below.

Reviewer 1: “I am unsure about the added value of reporting perplexity here. From my understanding, this metric is to some extent correlated with sequence recovery. Reporting both appears a bit redundant which is why I would propose to move Table 4 to SOM (or remove it altogether). Also perplexity depends on the vocabulary size which makes it even more complicated (potentially even misleading?) to compare Bert- with T5-based models on this metric within the same table (irrespective of proper PPL vs pseudo-PPL). “

We agree that perplexity correlates strongly with sequence recovery. We had initially provided it as it is a relatively standard metric for benchmarking language models, but we agree with the reviewer that providing both is somewhat redundant, and have decided to remove the table.

Reviewer 1: “Instead, you could consider adding some other prediction task (beyond affinity and expression) to make an even stronger point about whether the model-ranking provided in Table 3 holds true for other tasks as well. Although, I would understand that adding more benchmarks might be beyond the scope of this revision, so I would leave it to the authors whether to add this.”

Though it would be interesting to consider a broader set of downstream tasks, we found on datasets of less than several hundred data points there was not enough signal to fit models, with all models giving poor results and large variance across folds. We attempted to fit aggregation data from Flab, but did not see statistically meaningful results, possibly because the data is too noisy. The results on binding affinity and expression are the ones we found the most interesting to include from this analysis, one showing a slight performance improvement from our new models and the other showing that on certain tasks general protein language models achieve higher performance than antibody-specific ones.

Reviewer 1: “IgT5 saw only 700M samples, but I think this is not very well reflected in Fig. 2. Maybe try to make this more clear also throughout the text.”

We have updated the text to make it clearer than IgT5 has seen only 700M sequences (half of our unpaired OAS clusters).

Reviewer 1: “One potentially interesting way to bypass the computational complexity in the future might be to use some parameter-efficient-finetuning strategy s.a. LORA - this way you do not need to train all the parameters of the model which should allow you to increase throughput during training manifold (rather a suggestion for future improvement - I assume this also to be beyond this revision but I would be curious to see)”

We agree that more sophisticated fine-tuning strategies such as LORA would be a very good approach to specialize big pre-trained protein language models. To our knowledge there has been relatively little work in this direction in the biological space, and it is likely to open useful avenues for more careful ablation studies.

Reviewer 1: “Table 2: I am not sure how useful it is to report 4 digits after the comma. Consider only reporting e.g. 2 and instead add some error estimate as you did table 3.”

We have reduced the table to show only 3 digits. We do not show error estimates on this table as they are very small and make the table quite difficult to read.

Reviewer 1: “Binding affinity/expression: you write that the embeddings derived from the paired model had only half the size compared to the unpaired model. You make this very explicit which I highly appreciate but this also results in a model with only half the number of free parameters. As the paired model saw also unpaired samples during training (2/3 of the samples seen were unpaired if I understood correctly), I wonder whether you could add a line to Table 3 that shows the performance of embeddings derived from unpaired input given to your paired models and concatenated for input to the regression model (essentially, mimicking the process you ran for unpaired models but for paired models).”

We had experimented with using the same approach for the paired model as we did for the unpaired one, and average the token embeddings separately for each chain and then concatenate them. The dataset is too noisy to make conclusive statements and we found that this did not lead to a statistically significant change in performance. So we included the simpler approach in the paper of averaging over the full variable region embeddings, as we believe this is also one of the key advantages of paired antibody language models, to provide a coherent Fv-level representation of the antibody.

Reviewer 1: “In the discussion you write that “quality of paired data played a pivotal role in enhancing performance” but I am wondering where you show this?”

Our motivation for this statement is that sequence recovery has a plateau at about 92% recovery during the unpaired pretraining stage (as can be seen from table S3 on the unpaired test set), and during the first few epochs of training with paired data, the sequence recovery on paired data improves from a starting point of about 92% to about 94%. We interpret this gap in maximum possible recovery to quality of data, and notably sequencing errors that might be more common place in the high throughput single chain dataset. We have adapted this part of the discussion.

Reviewer 1: “Fig. 2: ProtT5 was not only pre-trained on UniRef50 but also on BFD (makes quite a difference as one has 2B sequences and the other has only tens of millions).”
We thank the reviewer for catching this oversight, and have updated the figure to reflect the full corpus of training sequences used.

Reviewer 2: “The authors utilized Linclust and MMseqs2 for approximate and exact clustering, respectively, both with a 95% sequence identity threshold. This threshold is crucial as it balances sequence diversity with redundancy, significantly affecting the construction of the training, test, and validation sets. The selection of this threshold influences the models' ability to generalize and perform effectively. It would be valuable for the authors to comment on and quantify how this threshold impacts the models' training efficiency and overall performance.”

We agree that the choice of sequence identity threshold is a key parameter choice that dictates the size and diversity of clusters seen during training. We used a threshold of 95% as a large fraction of the sequence corresponds to the framework region, and we are mostly interested in clustering based on small changes in the CDRs. Many other reasonable choices could have been made here, e.g. clustering only on concatenated CDR sequences or using a lower threshold to enforce higher diversity across clusters. Unfortunately, due to the very large computing costs associated with both the clustering stage (about a day on a 6 terabyte RAM computer) and the model training stage (14 days on 32 A100), it is impossible for us to provide ablation studies for different parameter choices, and it is hard to make conclusive statements about what the impact of different clusterings would be without being able to test them.

Reviewer 2: “Not surprisingly, the use of paired data improves the predictive performance across various tasks. However, the key question of how much paired data is necessary to achieve this performance boost is left unaddressed. Given that paired data is still relatively scarce, it would be valuable for the authors to provide performance metrics at various stages of fine-tuning on paired data. This could offer insights into the incremental benefits of paired data and help optimize its use in training.”

Though we have curves of the training loss which includes the MLM recovery rate on the training task and validation set, we do not have multiple checkpoints of the model at different training stages that we could evaluate on downstream tasks and on our final test set to make more quantitative statements about model performance on a range of tasks as a function of training data seen by the model. We agree that such a study would have been a valuable addition.

Reviewer 2: “The authors use batches containing both paired and unpaired data to prevent catastrophic forgetting, but they do not provide quantitative evidence to confirm whether catastrophic forgetting indeed occurs. To address this, similarly to the previous point, it would be beneficial to include performance metrics before, after, and at intermediate stages of fine-tuning on paired data, but now with a focus on tasks where unpaired data results in better performance (if any). This would allow for a clear assessment of the trade-off between fine-tuning on paired data and knowledge retention.”

Catastrophic forgetting is a common theme in fine-tuning ML models, and our assumption is that unless precautions are taken to mitigate it such forgetting will occur. We show in Table S3 that through our training strategy, the paired IgT5 and IgBert model achieve comparable

performance on a test set of unpaired sequences as the unpaired models, which in our view is evidence that the paired model retained knowledge of the unpaired sequence dataset. Internal preliminary tests performed on smaller scale model showed a substantial drop in performance (92% sequence recovery to down to 90%) in the paired model performance on the unpaired data when training without unpaired data in the final fine-tuning stage, but we do not have quantitative metrics that we can provide on the full scale model as we were only able to perform one training run. In Table 2, we show that the unpaired models do not perform as well as on a test set of paired sequences before being fine-tuned on paired sequences. Meanwhile, our benchmark on expression prediction shows that all our antibody language models have lost key information on general protein sequences that is useful to predict expression, as the original pre-trained ProtT5 and ProtBert models outperform the antibody specific ones on that task.

Reviewer 2: “If catastrophic forgetting does occur during, the authors could explore techniques to mitigate its effects. For instance, Elastic Weight Consolidation can penalize significant changes in weights critical for the original tasks, helping retain previously learned knowledge. Additionally, in progressive fine-tuning the model is gradually exposed to more paired data while intermittently retraining on unpaired data to preserve broader knowledge. These strategies would help balance the retention of general knowledge with the benefits of fine-tuning on paired data.”

We agree with this comment that applying techniques to prevent catastrophic forgetting could be an interesting alternative approach to using data from the previous training step. In particular, we think finding ways of retaining knowledge of general protein sequences would be an interesting area for future research. We have amended our discussion of catastrophic forgetting and added a citation to elastic weight consolidation.

Reviewer 2: “While the models excelled in antibody-specific tasks, they underperformed in predicting protein expression compared to general protein language models like ProtT5. The authors reason that antibody-specific models may have limitations in capturing the broader patterns necessary for accurate protein expression prediction, which are better learned by models trained on more diverse protein datasets. It would be valuable to investigate how general protein language models, such as ProtT5, perform on this benchmark when fine-tuned on antibody sequences. Indeed, while the authors demonstrate that models trained specifically on antibody sequences generally outperform general models, an important question left unexamined is how general models fine-tuned on antibody data would fare on this benchmark. Another important related question is whether extensive fine-tuning could cause general models like ProtT5 to lose the broad evolutionary knowledge that contributes to their superior performance in certain tasks (once again, catastrophic forgetting). If this is the case, it raises the critical issue of determining the optimal balance between retaining the acquired general knowledge and fine-tuning, and how this compares to a model entirely from scratch on antibody sequences. ”

Our IgT5 and IgBert models are fine-tuned from the ProtT5 and ProtBert model weights respectively. We agree that a more in depth study of different fine-tuning strategies and their

impact on generalizability of the models would be very valuable to the field, but unfortunately we do not have further computing resources to dedicate to this work at this time.

Reviewer 2: “This is particularly relevant given the high computational demands of training large language models from scratch (the authors used 32 A100 GPUs). Additionally, concerns about CO2 emissions and the importance of reducing redundant efforts further emphasize the need for optimizing the balance between training new models and fine-tuning existing ones.”

We agree that the environmental impact of training such large models should not be neglected, and our hope is also that by releasing our model, it provides a useful point of reference on training antibody language models at scale and avoids duplication costs.
