# Supplementary material for: Large scale paired antibody language models
Source: PLoS Comput Biol. 2024 Dec 6;20(12):e1012646. doi: 10.1371/journal.pcbi.1012646 (PMC11654935; doi:10.1371/journal.pcbi.1012646)
Supplement: Attachment — Submitted filename: reviewer_LMpaper.pdf [file pcbi.1012646.s006.pdf]

Reviewer 1: “I remain a bit skeptical/surprised about the comparatively small improvement after adding paired data but also such results are interesting. From their response, I understand that the authors made the claim about the improvement from unpaired to paired training data based on sequence recovery but a) I could not find the IgT5_unpaired=92% vs IgT5=94% reconstruction accuracy in Table 1 within SOM3 (if you are referring to the "total" in the rightmost column of the supplementary table, either my PDF viewer does not render it for the table 2 in main or you forgot to include this column/total in the main text table)”

Yes, we are referring to the total reconstruction accuracy in S3 Table 1, vs the total in Table 2. It is not clear to us why the table didn’t render correctly in the document the reviewer received, we hope it is readable in the latest version. In particular, for the light chain, IgT5-unpaired achieves 95.1% accuracy on paired VL sequences, while IgT5 achieves 96.6% accuracy. In comparison, on the unpaired test set, the accuracy is 92.9% for IgT5-unpaired and 92.7% for IgT5. So the performance that the model achieves is higher on paired sequences, and the gap between the paired and unpaired models on paired test data is noticeable.

Reviewer 1: “and b) even if I had found it, I would remain skeptical whether this improvement in reconstruction accuracy is meaningful. I think an improvement in reconstruction accuracy is a nice proxy but it only becomes really meaningful if it can be confirmed via better designs (probably the only use-case for reconstructing amino acids -but this most likely requires wet-lab testing) or if it correlates with better downstream prediction task quality (which I am not sure about after checking Table 3 in main). - All that being said: I appreciate that the authors re-phrased that they "believe" that paired data helps - I believe so, too, but apparently there remains some work to be done for fully leveraging this additional information. - Maybe make this a bit more transparent.”

We agree with the reviewer that our arguments are circumstantial evidence and that a more meaningful test would be to show quantitative improvement on designed sequences tested in a wet lab. We amended the discussion to reflect this.

Reviewer 1: “After cross-checking, the only thing that would be nice to add is the actual pre-training code (found only the huggingface entry with the weights and examples how to run the model but not the actual pre-training code).”

Unfortunately due to the constraints we face as industrial researchers, releasing the actual training code, which is coupled to internal tools, libraries and infrastructure, was not practically feasible. But our hope is that by releasing the model weights themselves as well as an easy way to run and fine-tune them through the huggingface library, this allows for full reproducibility of our study, as the model can be trained easily through existing huggingface training scripts, e.g. from the original ProtTrans study.

Reviewer #2: I'm disappointed with the responses. While I understand that some of my comments required substantial computational resources and may be beyond the scope of the revision, I expected the authors to at least acknowledge them as directions for future work or

open research questions. However, the response to my critique is reduced to just half a sentence in the Discussion. I strongly encourage the authors to improve this section by addressing these points more thoroughly.

We have extended the discussion section and incorporated additional suggestions from the initial review on the topics of clustering choices and performance scaling with paired data as avenues for future work. In the first revision, we had incorporated comments on catastrophic forgetting and fine-tuning.
In the original comment on our draft, reviewer #2 noted: “Indeed, while the authors demonstrate that models trained specifically on antibody sequences generally outperform general models, an important question left unexamined is how general models fine-tuned on antibody data would fare on this benchmark. Another important related question is whether extensive fine-tuning could cause general models like ProtT5 to lose the broad evolutionary knowledge that contributes to their superior performance in certain tasks”. This is in fact the core of what our study does: fine-tune a general protein language model on antibody data and show improved performance on certain tasks such as binding affinity prediction, while others such as expression, see a degradation in performance due to the fine-tuned models forgetting relevant evolutionary patterns. The comparisons with AntiBERTy and AbLang show that our models have similar patterns to models trained exclusively on antibody data, though they achieve higher performance, having been trained at much larger scale and on a much larger dataset. In our view one of the most interesting avenues for future work would be to incorporate general protein data into the second-stage training and study precisely how catastrophic forgetting of general protein knowledge, useful for expression or structure prediction, can be mitigated in large scale training of specialized models.
